# Performance comparison of first-order conditional estimation with interaction and Bayesian estimation methods for estimating the population parameters and its distribution from data sets with a low number of subjects

**DOI:** 10.1186/s12874-017-0427-0

**Published:** 2017-12-01

**Authors:** Sudeep Pradhan, Byungjeong Song, Jaeyeon Lee, Jung-woo Chae, Kyung Im Kim, Hyun-moon Back, Nayoung Han, Kwang-il Kwon, Hwi-yeol Yun

**Affiliations:** 1 College of Pharmacy, Chungnam National University, Daejeon, 34134 Republic of Korea; 2 Academic Planning Department, Yonsung Fine Chemicals, Suwon, 16675 Republic of Korea; 3 Department of Pharmacy, Faculty of Science, National University of Singapore, Singapore, 117543 Singapore; 4 College of Pharmacy, Korea University, Sejong, 30019 Republic of Korea; 5 College of Pharmacy, Chungnam National University, Daejeon, 305-764 South Korea

**Keywords:** Estimation methods, Few subjects, First-order conditional estimation with interaction, Markov chain Monte Carlo Bayesian, NONMEM

## Abstract

**Background:**

Exploratory preclinical, as well as clinical trials, may involve a small number of patients, making it difficult to calculate and analyze the pharmacokinetic (PK) parameters, especially if the PK parameters show very high inter-individual variability (IIV). In this study, the performance of a classical first-order conditional estimation with interaction (FOCE-I) and expectation maximization (EM)-based Markov chain Monte Carlo Bayesian (BAYES) estimation methods were compared for estimating the population parameters and its distribution from data sets having a low number of subjects.

**Methods:**

In this study, 100 data sets were simulated with eight sampling points for each subject and with six different levels of IIV (5%, 10%, 20%, 30%, 50%, and 80%) in their PK parameter distribution. A stochastic simulation and estimation (SSE) study was performed to simultaneously simulate data sets and estimate the parameters using four different methods: FOCE-I only, BAYES(C) (FOCE-I and BAYES composite method), BAYES(F) (BAYES with all true initial parameters and fixed *ω* ^*2*^), and BAYES only. Relative root mean squared error (rRMSE) and relative estimation error (REE) were used to analyze the differences between true and estimated values. A case study was performed with a clinical data of theophylline available in NONMEM distribution media. NONMEM software assisted by Pirana, PsN, and Xpose was used to estimate population PK parameters, and R program was used to analyze and plot the results.

**Results:**

The rRMSE and REE values of all parameter (fixed effect and random effect) estimates showed that all four methods performed equally at the lower IIV levels, while the FOCE-I method performed better than other EM-based methods at higher IIV levels (greater than 30%). In general, estimates of random-effect parameters showed significant bias and imprecision, irrespective of the estimation method used and the level of IIV. Similar performance of the estimation methods was observed with theophylline dataset.

**Conclusions:**

The classical FOCE-I method appeared to estimate the PK parameters more reliably than the BAYES method when using a simple model and data containing only a few subjects. EM-based estimation methods can be considered for adapting to the specific needs of a modeling project at later steps of modeling.

**Electronic supplementary material:**

The online version of this article (10.1186/s12874-017-0427-0) contains supplementary material, which is available to authorized users.

## Background

Exploratory preclinical (as well as clinical) trials may involve a low number of subjects (around 6 subjects). This is because in the early stages of drug development, statistical approaches are difficult to apply, potentially leading to bias when predicting population mean and distribution of parameters and/or all sources of variability. In addition, different aspects of the study design are not considered when calculating the number of subjects. As a result, it can be difficult to calculate and analyze the pharmacokinetic (PK) parameters, especially if the PK parameters show very high inter-individual variability (IIV).

Population analysis is a set of statistical techniques that can be used to study the average response (clinically measured event of any biomarker) in a population, as well as the IIVs in responses arising from different sources [1]. NONMEM is the gold standard software for population analysis that allows for mixed-effect modeling of PK/pharmacodynamic data while accounting for both unexplained inter-subject, inter-occasion, and residual variability (random effects), as well as measured concomitant effects (fixed effects). It can also be useful for analyzing data obtained from a low number of subjects involved in a study [2]. A list of estimation methods is available in NONMEM, including classical estimation methods [first-order conditional estimation with interaction (FOCE) and second-order approximation (LAPLACE)] and maximum likelihood expectation maximization (EM)-based estimation methods [iterative two-stage (ITS), important sampling EM (IMP), important sampling EM assisted by mode a posterior (IMPMAP), stochastic approximation expectation maximization (SAEM), and Markov chain Monte Carlo Bayesian (BAYES)]. Therefore, it is important to understand the performance of different approach-based methods for handling data with a low number of subjects.

Classical estimation methods like FOCE-I, including FO, FOCE and Laplace, approximate the likelihood by taking Laplace transformation and Taylor linearization [3]. These methods are known to perform well when models structure are simple and low in dimension. Here, the model with higher number of random-effect parameters (IIVs) are referred as of high dimensions. Furthermore, the classical estimation methods known to provide highly reproducible values, and short run-times for simple PK models [4]. However, these linearization methods fail to converge and estimate parameters precisely with significant bias with increase in model complexity. The EM based methods calculate the exact likelihood (with approximation) by sampling and summing through the probability density function space, which is theoretically expected to approach the true likelihood as the sampling reaches infinity. It is due to this sampling step EM based methods have longer run-time compared to the classical methods for simple PK models [5]. In case of complex PK/PD problems, EM based methods are faster than FOCE-I due to their efficient maximization step [4].

Some previous studies have compared available estimation methods with different objectives, identifying various desirable traits of estimation methods. The most desirable property of a given estimation method is its precision and accuracy as they are the basis of the reliability of the obtained estimates. Other expected features of the estimation methods are low sensitivity to priors and short runtime. However, no previous study has compared estimation methods for estimating population PK parameters from a small number of subjects. Therefore, the objective of this study was to compare precision and accuracy of estimation methods for estimating population mean and distribution of PK parameters from a small number of subjects and explore options to minimize bias with a classical method and a maximum likelihood EM-based method.

## Methods

**Fig. 1:**
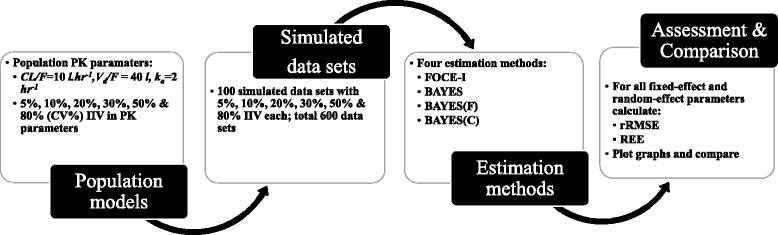
Scheme of the study design. Different steps of the study are outlined in the figure

An outline of this study is provided in Fig. 1; details are given in the following subsections. In this study, 100 data sets were simulated with eight sampling points for each subject and with six different levels of IIV (5%, 10%, 20%, 30%, 50%, and 80%) in their PK parameter distribution. The main reason for creating data sets was to describe close to real situations and minimize potential data set-dependent bias.

### Stochastic simulations and estimations

A stochastic simulation and estimation (SSE) study was performed using a one-compartment PK model. The estimation options in the model were varied to assess the performance of a classical estimation method – FOCE with the interaction option (FOCE-I), which allows for interaction between IIV(*η*) and residual variability(ε), and an EM-based estimation method – BAYES estimation method in NONMEM version 7.3.0 [6] assisted by Pirana (ver. 2.9.0), PsN (ver. 4.2.0), and Xpose (ver. 4.4.1) [7]. For statistical analysis of the results and generating different plots of the results, R (ver. 3.1.3) program was used [8].

### Population model and simulated data sets

The population model, specifically a one-compartment open model with first-order absorption and elimination rate constants, was used for simulation and estimation. The model consisted of three systematic PK parameters as fixed effects describing the absorption rate constant (*K*_*a*_), apparent volume of distribution (*V*_*d*_ */F*), and apparent clearance (*CL/F*), two random-effect parameters (*η*) describing the IIV on *V*_*d*_ */F* and *CL/F* [Eqs. (1, 2 and 3)], and a proportional error (ε) model (Eq. 4):

1 $$ {K}_a={\theta}_{K_a}, $$

2 $$ {V}_d/F={\theta}_{V_d/F}\bullet {e}^{\eta_{V_d/F}}, $$

3 $$ CL/F={\theta}_{CL/F}\bullet {e}^{\eta_{CL/F}}, $$

4 $$ {C}_{ij}={C}_{pred, ij}\left(1+{\varepsilon}_{ij}\right), $$

where C_ij_ indicates the *j-*th observations of *i-*th individual, C_pred, ij_ indicates the model-predicted C_ij_, and ε_ij_ indicates the proportional residual error.

The following equations [Eqs. (5) and (6)] describe the rate of change in drug amount in a one-compartment system:

5 $$ \frac{dA_d}{dt}=-{K}_a{A}_d, $$

6 $$ \frac{dA_c}{dt}={K}_a{A}_d-\frac{CL/F}{V_d/F}\ {A}_c, $$

where *A*_*d*_ and *A*_*c*_ are the drug amounts in the depot and central compartments, respectively, and *t* denotes the time.

The data set used for simulation consisted of six individuals with eight sampling points within 24 h after dosing for each individual. The population mean of PK parameters were assumed to be 2 L/h, 40 L, and 10 L/h for *K*_*a*_ *, V*_*d*_ */F*, and *CL/F*, respectively and their IIV levels (variance parameter ω^2^) were assumed to be 5%, 10%, 20%, 30%, 50%, and 80% coefficient of variance (CV%) (Eq. 7).

7 $$ CV\left(\%\right)=\sqrt{e^{\omega^2}-1}\times 100\%. $$

Data sets were simulated 100 times for each level of IIV (total of 600 data sets) and tested to compare estimation performance in NONMEM.

### Estimation methods

The population model was fitted to each of the simulated data sets using estimation methods with different estimation options and open or fixed ω^2^ values, as summarized in Table 1.

**Table 1:** Estimation methods and their conditions for initial parameters and estimation options

	Estimation method			
Methods	FOCE-I	BAYES(C)	BAYES(F)	BAYES
	First-order conditional estimation with interaction	FOCE-I and BAYES composite method	BAYES with ω^2^ value fixed to true value	Markov chain Monte Carlo Bayesian
Conditions				
Initial parameters	THETAs & OMEGAs: Open true values	THETAs & OMEGAs: Open true values	THETAs: Open true values OMEGAs: Fixed true values	THETAs & OMEGAs: Open true values
Estimation options	SIG = 3	*For FOCE-I,* SIG = 3 *For BAYES,* CTYPE = 3 NBURN = 4000 NITER = 10,000 SIGL = 8 NSIG = 2	CTYPE = 3 NBURN = 4000 NITER = 10,000 SIGL = 8 NSIG = 2	CTYPE = 3 NBURN = 4000 NITER = 10,000 SIGL = 8 NSIG = 2

The FOCE-I method is a classical estimation method that is applied by most users and has a short run-time for estimation of population mean and distribution for simple models [9, 10]. The BAYES method is a newly introduced method in NONMEM and is more suitable for estimation of population mean and distribution for complex PK/PD models [10]. In this study, the other estimation methods such as ITS, IMP, IMPMAP, and SAEM were not tested because these methods were expected to perform similar or below the performance of BAYES as these methods are based on EM algorithms. EM algorithms consist of an expectation (E) and a maximization step (M), where these methods differed in the way step E was performed, which involves the approximation of likelihood. Additionally, the BAYES method creates a large sample of probable parameters, unlike other EM-based methods that attempt to obtain a single “most likely” set of estimates.

In this study, true parameter values, i.e., the parameter values used in the simulation step, were established as initial estimates in all estimation methods. In NONMEM, convergence criteria for a FOCE-I are based only on the parameter estimation gradient and are tested by default. The number of significant digits for the estimation of each parameter was set to three (SIG = 3) for the FOCE-I method. In the BAYES estimation method, the convergence test type was set to 3 (CTYPE = 3), where changes in objective function value, THETAs, OMEGAs, and SIGMAs, are accessed. The number of significant digits to which the objective function was evaluated was set to 8 (SIGL = 8). In the BAYES methods, the maximum number of iterations for which to perform the burn-in phase was set to 4000 (NBURN = 4000), and the number of iterations for which to perform the stationary distribution for BAYES analysis was set to 10,000 (NITER = 10,000), both of which are default values in NONMEM. The former option ensured that all parameters and objective functions did not appear to move in a specific direction, but appeared to instead move around a stationary region, and the latter provides a large set (10,000) of likely population parameters.

### Assessment and comparison of estimation methods

The estimation methods were assessed by relative root mean squared error (rRMSE) and relative estimation error (REE) for fixed-effect as well as random-effect parameters to calculate and visualize the magnitude of differences between the true value and the estimated value. The rRMSE [Eq. (8)] provides a combined measure of bias and precision.

8 $$ rRMSE=\sqrt{\frac{\sum \frac{{\left({P}_{est}-{P}_{true}\right)}^2}{P_{true}}}{n^2}} $$

where *P*_*est*_ is the estimated parameter value, *P*_*est*_ is the true parameter values used at the simulation step, and *n* is the number of simulations for each set of *P*_*true*_ (*n* = 100).

REE was calculated [Eq. (9)] and plotted as box plots; the plot represents the relative bias by the median of the REE values and precision by distribution of REE about zero.

9 $$ REE=\sqrt{\frac{P_{est}-{P}_{true}}{P_{true}}.} $$

### Case study

The THEO data set available in the NONMEM distribution media was used as a case study. The estimated PK parameters and IIV from the final model fitted to the THEO data (called THEO model hereafter) was considered to be the population (true) mean values for PK parameters and IIV. SSE was performed using the THEO model, where 100 data sets were simulated from the model with six individuals in each data sets and four different estimation methods were used, listed in Table 1, to estimate the PK parameters and their IIV from the 100 data sets.

## Results

**Fig. 2:**
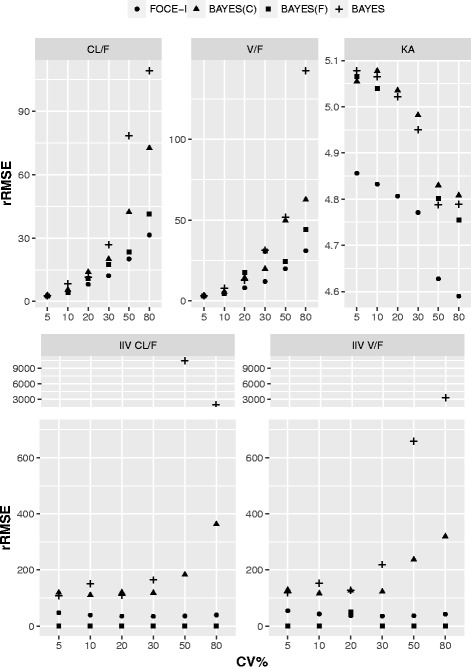
rRMSE plot for simulated data sets with 10%, 20%, 30%, 50% and 50% inter-individual variability. Relative root mean square error (rRMSE) of fixed-effect and random-effect parameters from simulated data sets with 10%, 20%, 30%, 50% and 50% inter-individual variability using FOCE-I (●), BAYES(C) (▲), BAYES(F) (■) and BAYES (┼) estimation methods

The rRMSE values of the estimated parameters (fixed-effect and random-effect) versus the level of IIV, stratified based on the different PK parameters, are shown in Fig. 2. The scale for each of the plots are adjusted to include all values. The analysis of all parameter rRMSE values showed that all four tested estimation methods performed equally at the lower IIV levels (5–30%), while the performance degraded with an increase in IIV. The FOCE-I method performed better than the other three EM-based estimation methods; this was more apparent at higher IIV levels (above 30%) for both fixed-effect and random-effect parameters. Performance of both the BAYES(C) and BAYES methods were poor at an IIV greater than 30% in terms of rRMSE. All parameter estimates at 50% and 80% IIV had exceptionally high rRMSE. The BAYES(F) performance was intermediary between FOCE-I and BAYES(C)/BAYES estimation methods in terms of rRMSE.

**Fig. 3:**
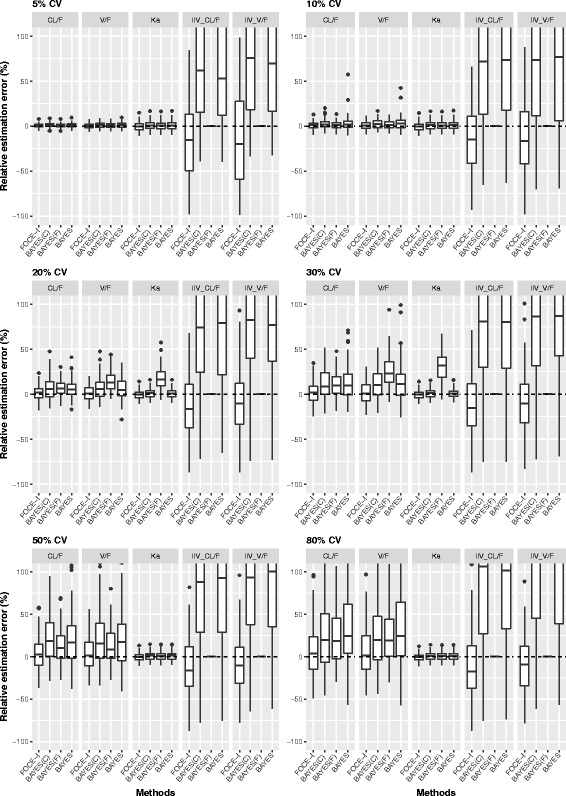
REE box-plot for simulated data sets with 10%, 20%, 30%, 50% and 50% inter-individual variability. Box-plot of relative estimation error (REE) of fixed-effect and random-effect parameters from simulated data sets with 10%, 20%, 30%, 50% and 50% inter-individual variability using FOCE-I, BAYES(C), BAYES(F) and BAYES estimation methods

The REE of both fixed-effect and random-effect parameters versus the estimation methods, stratified by different levels of IIV, are shown in Fig. 3. The plots were adjusted to include ±100% REE for the purpose of clarity. In general, all estimation methods overestimated fixed-effect parameters to some extent. At a lower level of IIV (5–10%), all estimation methods estimated fixed-effect parameters with negligible bias and reasonable precision. However, the bias as well imprecision increased with an increase in IIV variability. Overall, FOCE-I estimated fixed-effect parameters with REE near zero at all tested levels of IIV, while the distribution of REE increased with an increase in IIV. The other remaining three methods, BAYES(C), BAYES(F), and BAYES, had comparatively higher REE with a wider distribution range compared with the FOCE-I method.

The estimation of random-effect parameters had pronounced bias and imprecision, irrespective of the estimation method used or the level of IIV (with the exception of the BAYES(F) method, where the variance parameter was fixed to the true value) as shown in Fig. 3. Both EM-based methods, BAYES(C) and BAYES, performed poorly with higher bias and impression. Across all tested levels of IIV, BAYES(C) and BAYES methods had high bias and precision with skewed distribution of positive REE. The FOCE-I method consistently performed better compared with other methods with much lower and slightly negative bias where the distribution of REE overlapped with the zero value.

The overall stability of estimations were high with a 100% success rate of minimization and covariance step for BAYES(C), BAYES(F), and BAYES methods. For the FOCE-I method, the minimization step had a 100% success rate, but the rate of the successful covariance step was 52% at 5% IIV while other estimations had a successful covariance step close to 100%.

The THEO data set used as a case study had 132 observations from 12 subjects, 11 observations per individual after an oral dose of 320 mg theophylline. A one-compartment PK model with first order absorption described the data well and it was used as a final model. The PK parameters from the THEO data set were: *CL/F* = 2.88 l/h, *V*_*d*_ */F* = 33.01 l and *k*_*a*_ = 1.46 1/h and IIV were 25.69%, 13.48% and 65.39%, respectively. The rRMSE plots (Additional file 1) of the PK parameters from the THEO model show that the performance of the four estimation methods were similar for estimates of *CL/F* and *V*_*d*_ */F* both of which had lower IIV, below 30%. Whereas, overall higher rRMSE for estimate of *K*_*a*_ was observed, particularly from EM based methods. The estimation methods followed similar pattern of performance as indicated by rRMSE for estimation of random effect parameters. Similarly, REE box plots (Additional file 2) for estimated PK parameters show that *CL/F* and *V*_*d*_ */F* estimated by all four estimation methods were very close to the true values, where both of them had true IIV below 30%. For the estimate of *K*_*a*_, FOCE-I method estimated values were closes to the true value while estimated values from other three EM based method were positively biased (median REE above 25%) with low precision. Estimation of random effect parameters were poor for all the estimation methods, but the FOCE-I method performed relatively better in terms of bias and precision.

## Discussion

For an estimation method, the most desirable features are a low bias and high precision. In this study, we used rRMSE and REE to evaluate these features. The rRMSE provides a single value that indicates both bias and precision. Moreover, rRMSE provides a way to compare performance across parameters and models. However, the REE allows for comparison of different parameters with varying magnitudes in a single plot while acknowledging bias and precision. For an estimation method to be unbiased and precise, the REE should have a normal distribution with a median of 0 and a narrow range of values.

The FOCE-I method performed better among the four methods tested based on the overall rRMSE. This performance was supported by the REE plot, which did not show any significant bias for any fixed effect parameters at any given level of IIV. The median REE values for the random-effect parameters were not greater than −17% at any given level of IIV. A resembling result of negative bias was observed with the FOCE-I algorithm in a similar studies comparing different estimation methods [9]. The FOCE-I method has been shown to work sufficiently well for simple models when compared to other EM based algorithms in previous studies. Furthermore, when the IIV was low, the performance of classical estimation methods and EM based methods were very close. Similar results were observed in a previous study for such simple model (1-compartment model), where the performance of those estimation methods were found to be nearly equal [5].

On the other hand, rRMSE values for the three BAYES-based methods were significantly higher for both fixed- and random-effect parameters at higher levels of IIV. The higher rRMSEs were due to the wider spread of outliers, more so at higher levels of IIV. A similar trend of rRMSE of estimated parameters was observed using BAYES methods by Johansson et al., where the highly distorted rRMSE rendered the estimated parameters meaningless [9]. The performances of the BAYES-based methods were poor, with high bias and low precision. Even with the utilization of true values for all initial parameters, the BAYES(F) method was not able to estimate parameters close to the true values. Similarly, the median REE for all three methods based on the BAYES-method was comparatively higher for fixed-effect parameters and significantly higher for random-effect parameters, compared with those of the classical FOCE-I method. There was also a general trend of an increase in REE (positive) with an increase in IIV. These observations with BAYES-based methods can be attributed to the way in which the BAYES method estimates the parameters i.e., by generating a large set of probable population parameters and variance parameters that represent the distribution according to their ability to fit the data [11]. Therefore, the limited number of subjects used in the study may be the reason for the poor performance of the three BAYES-based methods. However, a previous study showed that the BAYES method can provide robust estimates of complex PK/PD models with rich data and reliable priors [10].

The classical estimation method, FOCE-I, and maximum likelihood EM-based BAYES method differ in their convergence criteria, where the former is based on changes in the parameter estimation gradient and are tested by default, and the latter is based on changes in objective function value and parameter estimates. The BAYES method can also define the convergence test type, and one can choose from no test, tests accessing changes in objective function, thetas and sigmas only, the addition of diagonals of omegas, or the addition of all omegas. For these reasons, the convergence rate was not included as a factor for comparison of estimation methods. However, all four methods tested at any level of IIV showed a 100% convergence rate. Additionally, in all estimation methods, the default or generally used values were used for options in the $ESTIMATION block. It is possible to optimize the outcomes by changing the values for different options in $ESTIMATION block [4]. However, this aspect of the estimation method was not compared, as this study only explored the practice of most users.

In this study, FOCE-I, the classical method, performed better with lower bias and higher precision compared with other BAYES-based methods. Moreover, the FOCE-I method is known to have a shorter run time that any other new methods [9, 12]. The work presented here compares a classical estimation method, FOCE-I, and BAYES method, with different options in the $ESTIMATION block and fixed OMEGA values (BAYES(C), BAYES(F), and BAYES) for population analysis of data with a low number of subjects (*n* = 6). Moreover, the models built had only one compartment, with basic PK parameters and random effects on two PK parameters. Therefore, it should be noted that the structure and complexity of a model might vary (increase or decrease) in different pharmacometric projects or within the same project from the initial to final step. In contrast to our study, other studies have shown that for complex models with highly non-linear functions [12], highly skewed count distributions [13, 14], and/or low variability or very rare events [15], the classical methods exhibit marked bias and impression. Additionally, the selection of an estimation method for a particular modeling project can depend on various aspects including bias, precision, robustness, runtime, data type, timeframe of project, application of results etc. which are objective in nature as well as subjective aspect such as preference for particular estimation method based on knowledge and previous experience. Ultimately, a pharmacometrician needs to make a choice for an estimation method based on multiple aspects.

The data sets used in this study, unlike real clinical data, were simulated. IIV for all PK parameters were assumed to be the same for an individual; i.e., IIV was either 5%, 10%, 20%, 30%, 50%, or 80% for *K*_*a*_ *, CL/F*, and *V*_*d*_ */F*. In clinical scenarios, the CV may vary widely among the PK parameters within an individual. Therefore, to access the relevance of results obtained from simulated data, a clinical data of theophylline involving 12 subjects, THEO data set, was used as a case study. The limitation of using real data is that the expected true parameters value is unknown. So, SSE was performed, where the parameter estimates from final THEO model was considered to be true parameters. And the parameter estimates from different estimation methods were compared to so-called true values for compare their performance. Similarity in the performance of all four estimations methods at lower IIV and better performance of FOCE-I methods at higher IIV was demonstrated by the rRMSE and REE of the estimated parameters. This further supports the results from the simulated data. Another limitation of this study is that only FOCE-I and BAYES methods were tested and compared. To further explore the best estimation method when dealing with a low number of subjects, other methods in NONMEM, such as LAPLACE, ITS, IMP, IMPMAP, and SAEM should also be evaluated in future studies.

## Conclusions

The FOCE-I, a classical estimation method, yielded better results in terms of bias and precision across all levels of IIV in comparison to three variations of BAYES estimation methods. The difference in performance between FOCE-I and three BAYES estimation methods in estimating fixed-effect parameters were significant only at the IIV level greater than 30%. The bias and imprecision of random-effect parameters were higher compared with fixed-effect parameters, however, it was consistently lower for FOCE-I method compared to those estimated using BAYES(C) and BAYES methods. These results were further supported by the results from the THEO data, where clinical data was used to simultaneously simulate and estimate PK parameters using FOCE-I and three BAYES estimation methods.

In conclusion, the classical FOCE-I method estimated the PK parameters more reliably than the BAYES method when using a simple model and data containing only a few subjects. After the base modeling step is complete and/or at the pivotal modeling step, use of other EM-based estimation methods can be considered for adapting to specific needs of the project.

## Additional files

Additional file 1: rRMSE plot for THEO data set. Relative root mean square error (rRMSE) of fixed-effect and random-effect parameters from THEO data set using FOCE-I (●), BAYES(C) (▲), BAYES(F) (■) and BAYES (┼) estimation methods. (PDF 6 kb)

Additional file 2: REE box for THEO data set. Box-plot of relative estimation error (REE) of fixed-effect and random-effect parameters from THEO data set using FOCE-I, BAYES(C), BAYES(F) and BAYES estimation methods. (PDF 8 kb)

## Abbreviations

BAYES: Markov chain Monte Carlo Bayesian
BAYES(C): First-order conditional estimation with interaction and Markov chain Monte Carlo Bayesian composite method
BAYES(F): Markov chain Monte Carlo Bayesian with variance parameter fixed to true value
CL/F: Apparent clearance
Ɛ: Residual variability
EM: Expectation maximisation
FOCE: First-order conditional estimation
FOCE-I: First-order conditional estimation with interaction
IIV: Inter-individual variability
IMP: Important sampling
IMPMAP: Important sampling assisted by mode a posterior
ITS: Iterative two-stage
Ka: Absorption rate constant
ƞ: Inter-individual variability
PK: Pharmacokinetic
REE: Relative estimation error
rRMSE: Relative root mean square error
SAEM: Stochastic approximation expectation maximization
SSE: Stochastic simulation and estimation
Vd/F: Apparent volume of distribution

## References

[1] Duffull SB, Wright DF, Winter HR (2011). Interpreting population pharmacokinetic-pharmacodynamic analyses - a clinical viewpoint. Br J Clin Pharmacol.

[2] Mould DR, Upton RN (2013). Basic concepts in population modeling, simulation, and model-based drug development-part 2: introduction to pharmacokinetic modeling methods. CPT Pharmacometrics Syst Pharmacol.

[3] Wang Y (2007). Derivation of various NONMEM estimation methods. J Pharmacokinet Pharmacodyn.

[4] Gibiansky L, Gibiansky E, Bauer R (2012). Comparison of Nonmem 7.2 estimation methods and parallel processing efficiency on a target-mediated drug disposition model. J Pharmacokinet Pharmacodyn.

[5] Liu X, Wang Y (2016). Comparing the performance of FOCE and different expectation-maximization methods in handling complex population physiologically-based pharmacokinetic models. J Pharmacokinet Pharmacodyn.

[6] Beal SL, Sheiner LB, Boeckmann AJ, Bauer RJ. NONMEM 7.3.0 User Guides. ICON Dev Solut. 2013;

[7] Keizer RJ, Karlsson MO, Hooker A (2013). Modeling and simulation workbench for NONMEM: tutorial on Pirana, PsN, and Xpose. CPT Pharmacometrics Syst Pharmacol.

[8] R Development Core Team (2014). R: a language and environment for statistical computing.

[9] Johansson AM, Ueckert S, Plan EL, Hooker AC, Karlsson MO (2014). Evaluation of bias, precision, robustness and runtime for estimation methods in NONMEM 7. J Pharmacokinet Pharmacodyn.

[10] Bauer RJ, Guzy S, Ng C (2007). A survey of population analysis methods and software for complex pharmacokinetic and pharmacodynamic models with examples. AAPS J.

[11] Bauer RJ. Technical guide on the expectation-maximization population analysis methods. ICON Dev Solut. 2013;

[12] Plan EL, Maloney A, Mentre F, Karlsson MO, Bertrand J (2012). Performance comparison of various maximum likelihood nonlinear mixed-effects estimation methods for dose-response models. AAPS J.

[13] Plan EL, Maloney A, Troconiz IF, Karlsson MO (2009). Performance in population models for count data, part I: maximum likelihood approximations. J Pharmacokinet Pharmacodyn.

[14] Savic R, Lavielle M (2009). Performance in population models for count data, part II: a new SAEM algorithm. J Pharmacokinet Pharmacodyn.

[15] Karlsson KE, Plan EL, Karlsson MO (2011). Performance of three estimation methods in repeated time-to-event modeling. AAPS J.

