# Supplementary figures and images for: Performance comparison of first-order conditional estimation with interaction and Bayesian estimation methods for estimating the population parameters and its distribution from data sets with a low number of subjects
Source: BMC Med Res Methodol. 2017 Dec 1;17:154. doi: 10.1186/s12874-017-0427-0 (PMC5709938; doi:10.1186/s12874-017-0427-0)

● FOCE-I ▲ BAYES(C) ■ BAYES(F) + BAYES

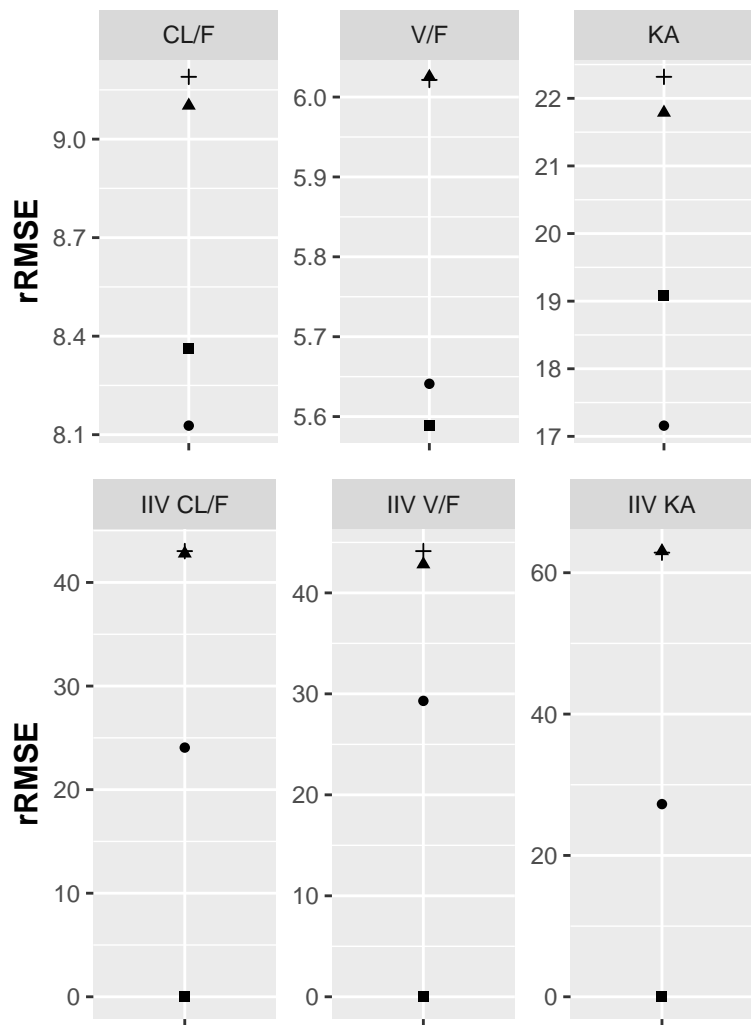

Supplement: Supplementary file 1 — rRMSE plot for THEO data set. Relative root mean square error (rRMSE) of fixed-effect and random-effect parameters from THEO data set using FOCE-I (●), BAYES(C) (▲), BAYES(F) (■) and BAYES (┼) estimation methods. (PDF 6 kb) [file 12874_2017_427_MOESM1_ESM.pdf]

## THEO data

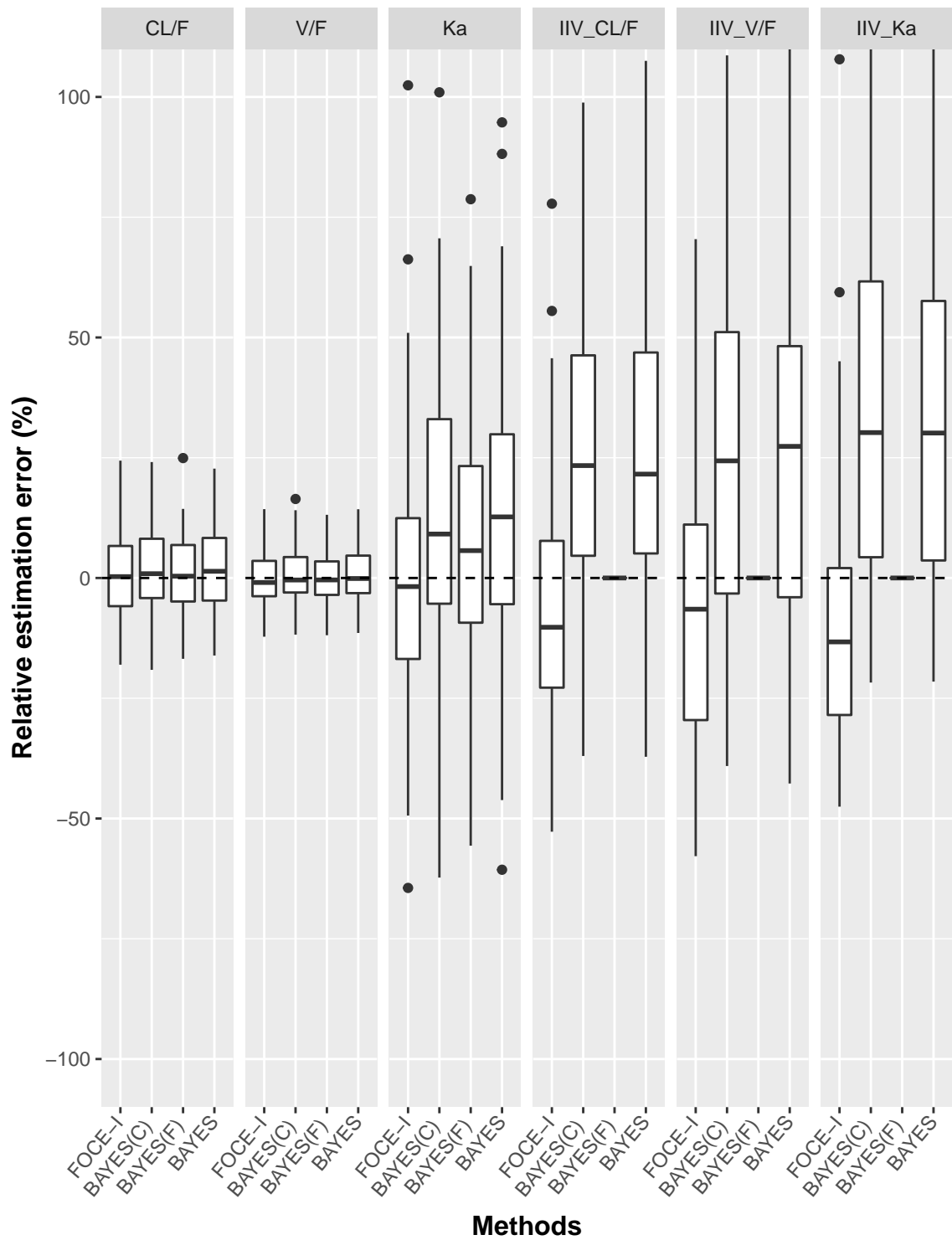

Supplement: Supplementary file 2 — REE box for THEO data set. Box-plot of relative estimation error (REE) of fixed-effect and random-effect parameters from THEO data set using FOCE-I, BAYES(C), BAYES(F) and BAYES estimation methods. (PDF 8 kb) [file 12874_2017_427_MOESM2_ESM.pdf]
